# Blood-nerve barrier enhances chronic postsurgical pain via the HIF-1α/ aquaporin-1 signaling axis

**DOI:** 10.1186/s12871-023-02306-7

**Published:** 2023-11-21

**Authors:** Pei-zhi Wu, Ju Yao, Bei Meng, Yi-Bin Qin, Su Cao

**Affiliations:** Department of Anesthesiology, Affiliated Hospital and Medical School of Nantong University, No. 20 Xisi Road, Nantong, 226001 Jiangsu China

**Keywords:** Chronic postsurgical pain, Dorsal root ganglion, Blood-nerve barrier, Hypoxia, Water transport

## Abstract

**Background:**

Blood nerve barrier (BNB) participates in the development of neuropathic pain. AQP1 is involved in peripheral pain perception and is negatively correlated with HIF-1α phenotype, which regulates endothelial permeability. However, the role of HIF-1α-AQP1-mediated BNB dysfunction in Chronic Postsurgical Pain (CPSP) has not been reported.

**Methods:**

Male Sprague-Dawley rats were randomized into 5 groups: (i) Naive group; (ii) Sham group; (iii) SMIR group: skin/muscle incision and retraction for one hour. Behavioral tests were performed for the three groups, BNB vascular permeability and western blotting were conducted to determine HIF-1α and AQP1 protein expression. (iv) The SMIR + HIF-1α inhibitor group; (v) SMIR + DMSO group. Rats in the two groups were administered with HIF-1α inhibitor (2ME2) or DMSO intraperitoneally on the third day post-SMIR surgery followed by performance of behavioral tests, BNB permeability assessment, and determination of HIF-1α, AQP1 and NF200 protein levels.

**Results:**

The permeability of BNB was significantly increased and the expression of AQP1 was downregulated on the 3rd and 7th days post-operation. AQP1 is mainly located in neurons and NF200, CGRP-positive nerve fibers. HIF-1α was highly expressed on the third day post-operation. HIF-1α inhibitor reversed the decrease in AQP1 expression and increase in NF200 expression, barrier permeability and hyperalgesia induced by SMIR on the 3rd day post-surgery.

**Conclusions:**

Early dysfunction of BNB mediated by HIF-1α/AQP1 activated by SMIR may be an important mechanism to promote acute postoperative painful transformation of CPSP. Preadaptive protection of endothelial cells around nerve substructures may be an important countermeasure to inhibit CPSP transformation. Early impairment of BNB function mediated by HIF-1α/AQP1 activated by SMIR may be an important mechanism for promoting acute postoperative pain transformation of CPSP.

**Supplementary Information:**

The online version contains supplementary material available at 10.1186/s12871-023-02306-7.

## Introduction

The blood nerve barrier (BNB) is a barrier system existing between the blood and the peripheral nervous system. It is a functional “neurovascular unit” formed by endothelial cells, glial cells and neurons, and regulates the stability of endoneurium microenvironment and the normal activity of neurons. Chronic destruction of its structure and function leads to irreversible increase in endoneural vascular permeability, which plays a key role in the development of inflammation and neuropathic pain after peripheral nerve injury [1–4], but its role in Chronic Postsurgical Pain (CPSP) has not been elucidated.

Aquaporins (AQPs) are distributed in various tissues, where they facilitate the bidirectional diffusion of water and small solutes across membranes. Moreover, they play a role in development of non-communicable diseases such as neurological disorders and metabolic syndrome [5]. Physiologically, AQP1 regulates tissue and water balance, and the multipotent property in biological activities, including inflammation and angiogenesis [6], and regulates peripheral pain perception [7]. AQP1 plays an important role in allodynia induced by chronic compression of dorsal root ganglion(DRG) [8]. Hypoxia-inducible factor 1 (HIF-1), an important regulatory factor in injury response, induces vascular permeability under stress and has neuroprotective effects [9]. Moreover, the stability of HIF-1α was increased under hypoxic conditions. HIF-1α promotes cell hypoxia adaptation through its target genes erythropoietin, vascular endothelial growth factor and glucose transporter [10–12]. Inhibition of the HIF-1α/VEGF axis is a potential approach for the treatment of acute bone cancer pain and rheumatoid arthritis pain [13, 14]. However, the interaction between HIF-1α and AQP1 in CPSP has not been reported.

Skin/muscle incision and retraction (SMIR) is a model of persistent postoperative pain caused by direct injury to the peripheral nerves, which better reflects the characteristics of inflammatory microenvironment in the surgical area, and induces CPSP through c-Raf and ERK1/2 [15]. SMIR activates microglia cells in the early stage and upregulates the levels of IL-1α and TNF-α in the spinal cord. The proinflammatory phenotype of astrocytes in the spinal cord was induced 14 days after SMIR, prompting CPSP [16]. In this experiment, the rat SMIR model was established as previously described [17] to observe the changes in BNB function, HIF-1α and AQP1 expression in DRG, and explore the role of BNB in early CPSP.

## Materials and methods

### Animals

Eight to ten-week-old male Sprague-Dawley rats (180-220 g) were purchased from the Laboratory Animal Center of Nantong University. Rats were allowed to acclimatize to the animal behavior laboratory (23 ± 1℃, humidity of 55–60%, and the cycle of light and dark lasts 12 h) for three days and supplied with water and food ad libitum. They were routinely fed post-surgery, and there were no wound infections, hair loss, diarrhea or other symptoms.

### Study groups

Rats were randomized into 5 groups: (i) Naive group: no treatment. (ii) Sham group: They were anesthetized with intraperitoneal administration of 30 mg/kg 3% pentobarbital sodium (Merck, Darmstadt) following by fixation in a supine position. Their skins were incised with a length of 1.5-2 cm at 3-4 mm from the saphenous vein on the medial side of the middle thigh of the right lower extremity, sutured under sterile conditions and covered with a sterile saline gauze. (iii) Experimental group (SMIR group): Similar to the sham operation group, the superficial leg muscles were exposed, the saphenous nerve was avoided, and the muscles were bluntly separated. Below the superficial muscles, the retractable device’s tip was positioned, stretched to 2 cm for 60 min, and sutured. (iv) The SMIR + HIF-1α inhibitor group (HIF-1α inhibitor group): The HIF-1α inhibitor (2ME2, 15 mg/kg, dissolved in 20% DMSO) (Selleck, USA) was injected intraperitoneally on the third day post-SMIR surgery. (v) SMIR + DMSO group: Post-SMIR surgery, an intraperitoneal injection of the same 20% DMSO solvent was injected on the third day.

### Establishment of postoperative persistent pain rat models

The 30 mg/kg 3% pentobarbital sodium was injected intraperitoneal into rats to anesthetize them and then fixed them in the supine position. Under sterile conditions, their skins were cut 1.5-2 cm at about 4 mm from the right thigh’s saphenous vein to expose the superficial muscle layer. Their skins were sliced about 7–10 mm, and the white adductor membrane was seen. A miniature retractor was placed below the muscle and the skin stretched to 2 cm for 1 h. During retraction, the saphenous nerve was displaced and not oppressed by the bone and other objects. The incision was covered with a sterile saline gauze for moisture and heat preservation. After operation, the incision was sutured layer by layer, and the wound condition was observed after operation.

### Paw withdrawal mechanical threshold (PWMT) detection

All assays in the test room were carried out between 9:00 and 11:00 a.m. by the same experimenter in a quiet environment, where temperature and humidity levels were stable. Before and on the 1st, 3rd, 7th, 14th, and 21st days following surgery, the PWMT was performed with the up-and-down method [18]. Rats were acclimated to the metal sieve mesh in a Plexiglas box (22 × 12 × 22 cm^3^) for 30 min. Then, with a logarithmic increase in rigidity (0.16-26 g), a stimulator made with Von Frey filament ciliary cords (North Coast Medical, USA) was used to vertically stimulate the middle part of the paw bottom of the hind limb on the surgical side of the rat in a calm state. Slight bending of the von Frey filament was used as the standard of complete force, the duration of which was ≤ 4 s. Retraction of the paw, shaking of the paw, lick of the paw, along with other phenomena, were deemed favorable reactions; in any other case, the response was regarded as unfavorable. For measurements, the lifting approach was employed beginning at 0.16 g, and the outcomes were recorded five times. If there were two or more positive reactions over the course of five, mechanical touch induced pain was considered to have been achieved. In case there were no more than 2 positive reactions, the adjacent high degree of intensity stimulation was initiated. In cases of more than 2 positive reactions, the adjacent low degree of intensity stimulation was initiated. This experiment was continuously performed until a positive or undesirable straddle response was observed, after which 5 consecutive measurements were performed at an interval of 30 s. According to the threshold table, we determined the claw retraction threshold, and “g” was used as the threshold unit. Mechanical hyperalgesia was more serious when the claw contraction threshold was lowered.

### Western blotting assay

Rats were deeply anesthetized by intraperitoneal injection of 3% sodium pentobarbital at a dose of 70 mg/kg and then 200 ~ 300 ml normal saline was injected into the hearts of rats. The L4-6 DRG tissues of the injured sides of rats were obtained. The skin and deep fascia were cut from the back of the rat, separating the muscles on both sides of the spine, exposing the sciatic nerve of the deep muscles. Trace the L4-6 branch retrograde along the sciatic nerve to the foramen, and pull the pale-yellow oval DRG out of the foramen along the direction of the nerve shape. After weighing, DRG tissues were supplemented with 1 ml of RIPA lysate and protease inhibitor per 100 mg of tissue. Subsequently, 1 ml of RIPA lysate and protease inhibitor were added to every 100 mg of tissue, and lysed on an ice surface for 10 min. After 30 min in ice, each tube was filled with 100 µl of supernatant. By centrifugation at 12,000 RPM for 15 min at 4 °C in a cryogenic high-speed centrifuge (Eppendorf, 5417R, Germany), total cellular proteins were extracted and stored at -80 °C. Protein concentrations were determined by UV spectrophotometry. SDS-polyacrylamide gel electrophoresis (PAGE) was performed on 5% concentrated glue and 10% separated glue (Epizyme). Each well contained 30 µg total protein and the required protein solution volume (µl) calculated as 30 µg protein/protein concentration. After electrophoresis, the protein was transferred to 0.45 μm PVDF membrane and at room temperature and then washed with TBST containing 5% skim milk powder for 2 h. The membrane was incubated with mouse anti-AQP1 antibody (1:200, Santa Cruz Biotechnology, USA), rabbit anti-HIF-1α (1:1000, Abcam, UK) and polyclonal antibody for NF-H/NF200 (1:200, Proteintech, China) at 4℃ overnight. Next, we diluted the secondary antibodies with PBS of 0.01 mol/L: HRP-goat anti-mouse IgG (1:5000, Proteintech, China) and HRP-conjugated Affinipure Goat Anti-Rabbit IgG (H + L) (1:3000, Proteintech, China), secondary antibodies were incubated with the membrane for two hours at room temperature. The membrane was washed and detected using the chemiluminescent substrate kit in a gel imaging system (Beijing Yuanpinghao Biotechnology Co., LTD., Tanon 2500). With β-actin as the internal reference, analyses were carried out using Image J software. The ratio of gray value of target proteins to that of reference protein was calculated to determine the relative protein expression.

### The BNB permeability test

After deep anesthesia, 2% Evans blue (Evan) solution was slowly injected at 2 ml/kg into the rats’ left femoral veins. After one minute, a blue coloration appeared on the skin of the toes, ears, and other areas, indicating success. After 60 min, the heart was perfused with 100 ~ 200 ml normal saline at 37℃, and a clear liquid flowed out 5 min later. The L4-6 DRG tissue was obtained and placed into the EP tube. Next, 2% EB was diluted 100 times, liquids with 20 ng/ug were diluted to prepare serial concentrations of 10, 5, 2.5, 1.25, 0.625, 0.313, 0.156 ng/ug with empty tubes of formamide, as a result, the standard curve was constructed. With a concentration of 100 mg/ml, a solution of 2% formamide was used to soak the tissue for homogenization, set the tissue in a chamber at 60 °C for 24 h, spent 15 min centrifuging it at high speed (11,000 rpm), after which, there were three tube samples for the supernatant collected and divided into each of them (40ul/tube). A measurement of absorbance (A) was made at 620 nanometers using the enzyme label instrument (BioTek, USA). Evan levels in the sample were calculated according to the regular curve, and Evan’s mean leakage outside of the blood vessel measured in ng/mg.

### Immunofluorescence staining

After deep anesthesia, rats were injected with normal saline and 4% paraformaldehyde through the heart. DRG tissues of rats were taken and placed in 4% paraformaldehyde overnight, then dehydrated in 20% and 30% sucrose solution, respectively, and embedded with OCT. The fixed tissues were sliced to a thickness of.

14 μm in a cryostat and stored in the refrigerator at -20 ° C. The frozen slices were obtained, baked in a 37℃ oven for 30 min, soaked in the PBS buffer at 0.01 mol/L for 10 min. Rinsed thrice in 0.01 mol/L PBS for 5 min each time. Subsequently, they were sealed for 2 h with 5% serum antibody at room temperature. The primary antibody diluted with 1% BSA was added drop by drop at 4 °C overnight. The primary antibody was diluted using the 2.5% serum antibody dilution. Mouse anti-AQP1 antibody (1:200, Santa Cruz Biotechnology, USA), rabbit anti-NeuN antibody (1:100, ABclonal Technology, USA), rabbit anti-CGRP antibody (1:500, Abcam, UK) and rabbit anti-NF200 antibody (1:500, Abcam, UK) were added to the sections by groups, and incubated overnight at 4℃ refrigerator. PBS of 0.01 mol/L was used to rinsed sections thrice for 10 min each time. We diluted the secondary antibodies with PBS of 0.01 mol/L: goat anti-mouse IgG H&L 488 (1:500, Abcam, UK) and goat anti-rabbit IgG H&L 594 (1:500, Abcam, UK). The corresponding secondary antibodies were added and then incubated with tissue sections for 2 h at room temperature in darkness. They were the mounted in darkness, dried and sealed using a fluorescent tablet sealer. A fluorescence microscope was used to observe and image portions (LEICA, Germany). The fluorescence film was obtained from the image analysis system, double labeled and analyzed.

### Statistical analysis

Data were analyzed using SPSS 20.0 software and expressed as the mean ± SEM. Two-way repeated measures ANOVA was used to compare behavioral data followed by the Bonferroni *post hoc* test for between-group comparisons. Specific band density and immunofluorescence intensity of Western Blot were evaluated with the Image J software. One-way ANOVA was used for comparisons of means among groups while the student’s t-test was used for comparison of means between groups. P < 0.05 denoted significant differences.

## Results

### PWMT was significantly reduced by SMIR, as shown in Fig. 1

The SMIR model was established, and the PWMT was determined using Von Frey filaments. The results showed that there was no significant difference in baseline values (BL) among the groups. There were no significant differences in PWMT between the Sham group and the Naive group at all time points post-surgery, while PWMT in the SMIR group was significantly decreased at the 1st, 3rd, 7th, 14th, 21st day post-surgery (*P* < 0.05, *P* < 0.01, *P* < 0.001), reaching the lowest value on the 7th day post-surgery. These findings implied successful establishment of the CPSP model.

**Fig. 1:**
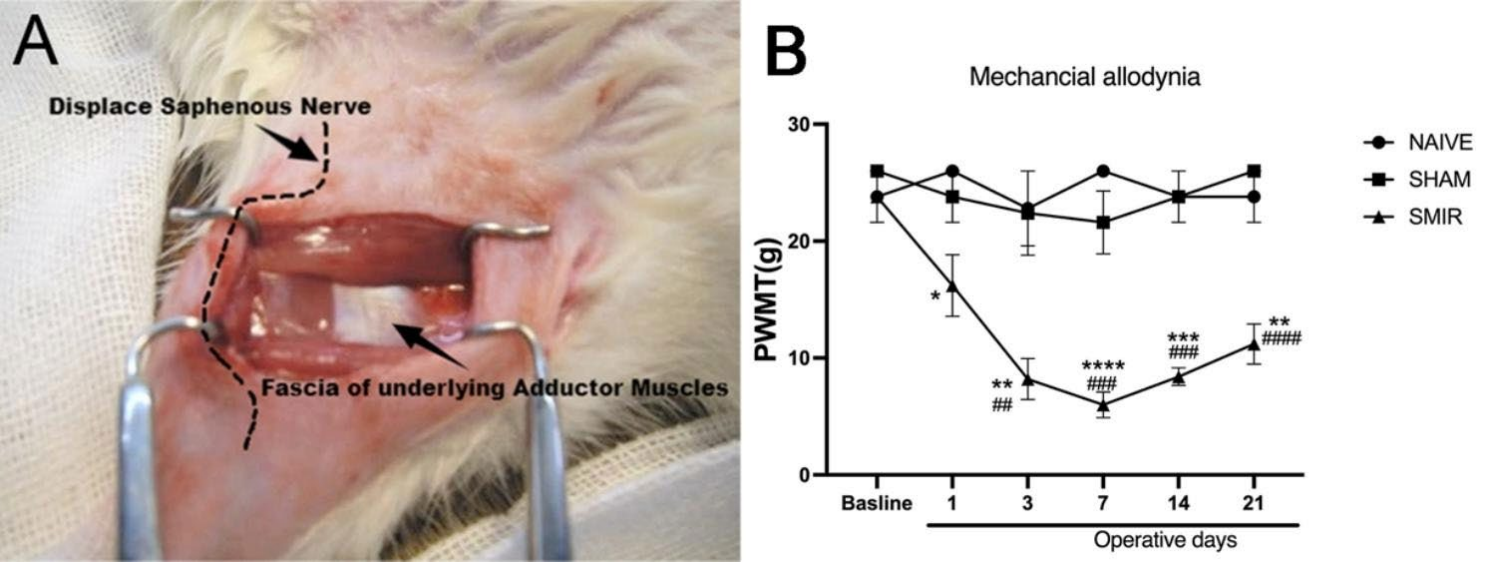
PWMT was significantly reduced by SMIR. **(A)** The SMIR model was established. **(B)** The PWMT of the SMIR model was determined using Von Frey filaments. The PWMT in the SMIR group was significantly decreased at the first, 3rd, 7th, 14th, 21st day post-surgery (*P < 0.05, **P < 0.01, ***P < 0.001, ****P < 0.0001 vs. naive group. ^##^*P <* 0.01, ^###^*P* < 0.001 vs. sham group; n = 6), reaching the lowest value on the 7th day post-surgery

### Effects of SMIR on BNB, AQP1 and HIF-1α levels in DRG, as shown in Fig. 2

Evans Blue was slowly injected into the femoral vein for BNB function test in DRG. The expression levels of AQP1 and HIF-1α were analyzed by Western-blot, showing: compared with the naive group, the amount of Evans blue exudation was significantly increased (*P* < 0.001) and the expression level of AQP1 was significantly decreased (*P* < 0.05) on the 3rd and 7th day post-surgery in the SMIR group, the expression level of HIF-1α was significantly increased (*P* < 0.05) on the 3rd day post-surgery in the SMIR group. There were no significant changes in Sham group. These results indicated that SMIR induced BNB dysfunction in DRG and promoted microvascular endothelial hyperpermeability. SMIR promoted HIF-1α expression and inhibited AQP1 expression in DRG.

**Fig. 2:**
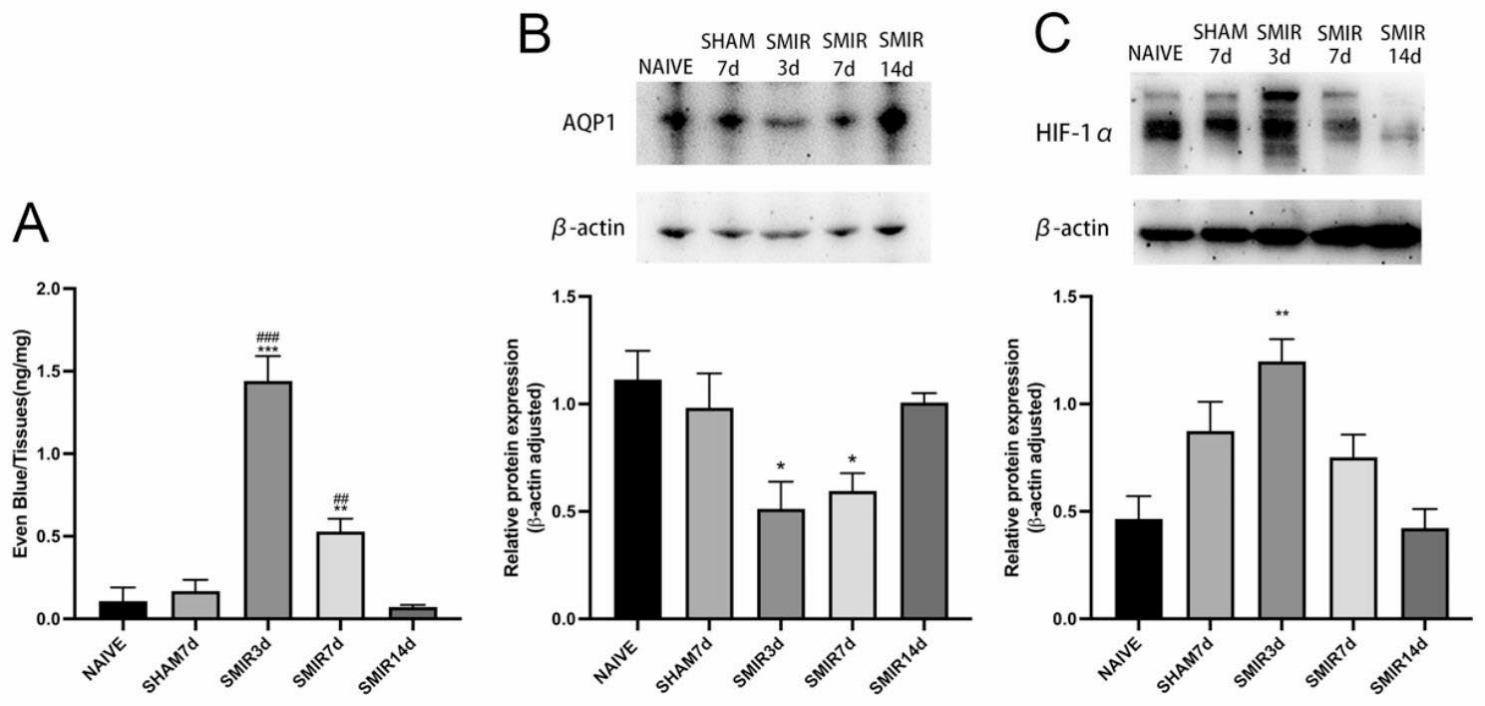
Effects of SMIR on BNB, AQP1 and HIF-1α levels in DRG. **(A)** SMIR induced BNB dysfunction in DRG and promoted microvascular endothelial hyperpermeability. The amount of Evans blue exudation was significantly increased on the 3rd and 7th day post-surgery in the SMIR group (**P < 0.01, ***P < 0.001 vs. naive group. ^##^*P* < 0.01, ^###^*P* < 0.001 vs. sham group; n = 5). **(B-C)***Changes of AQP1 and HIF-1α protein expression in DRG after SMIR.* SMIR promoted HIF-1α expression and inhibited AQP1 expression in DRG. On the 3rd and 7th days post-surgery, there was a marked decrease in AQP1 levels. HIF-1α protein expression was significantly elevated in the SMIR group on the 3rd day post-surgery (*P < 0.01 vs. naive group; n = 5)

### Localization of AQP1 in neuronal cells in DRG post-SMIR operation, as shown in Fig. 3

Immunofluorescence co-staining of AQP1 with the neuronal marker (NeuN) was performed on DRG tissue sections from the blank group and at the 3rd, 7th and 14th day after SMIR. The results showed that co-localization of AQP1 and neuronal marker NeuN increased on the 3rd and 7th day post-SMIR surgery compared with naive group, and the increase was obvious on the 3rd day post-surgery.

**Fig. 3:**
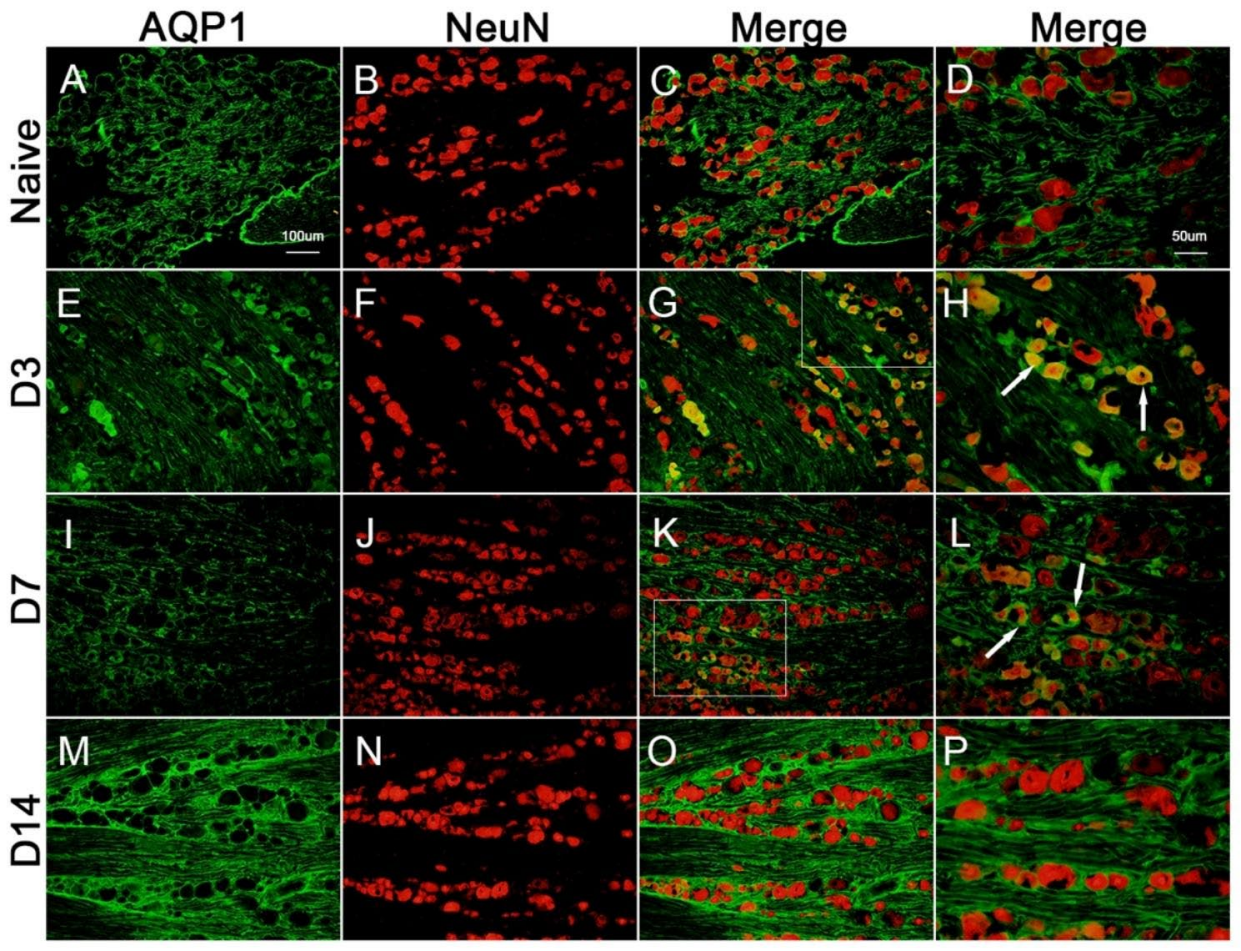
Immunofluorescence staining showed the localization changes of AQP1 in neurons before and after surgery. **(A-D)** There was no obvious co-labeling between AQP1 and neuronal marker NeuN in Naive group. **(E-H)** On the 3rd day post-surgery, the co-labeling of AQP1 and neuronal marker NeuN increased significantly. **(I-L)** The co-labeling of AQP1 and the neuronal marker NeuN increased significantly on the 7th day post-surgery. **(M-P)** There was no obvious co-labeling between AQP1 and neuronal marker NeuN on the 14th day post-surgery

### Localization of AQP1 in subneuronal cells in DRG post-SMIR, as shown in Fig. 4

Immunofluorescence co-staining of AQP1 with the peptide neuronal marker (CGRP) and axon marker (NF200) was performed on DRG tissue sections of the blank group and at the 3rd, 7th and 14th day after SMIR, showing that compared with naive group, co-localization of AQP1 and peptide neuronal marker CGRP increased on the 3rd and 7th day post-SMIR surgery, and the increase was obvious on the 3rd day post-surgery. Co-localization of AQP1 and axon marker NF200 increased on the 3rd day post-SMIR surgery.

**Fig. 4:**
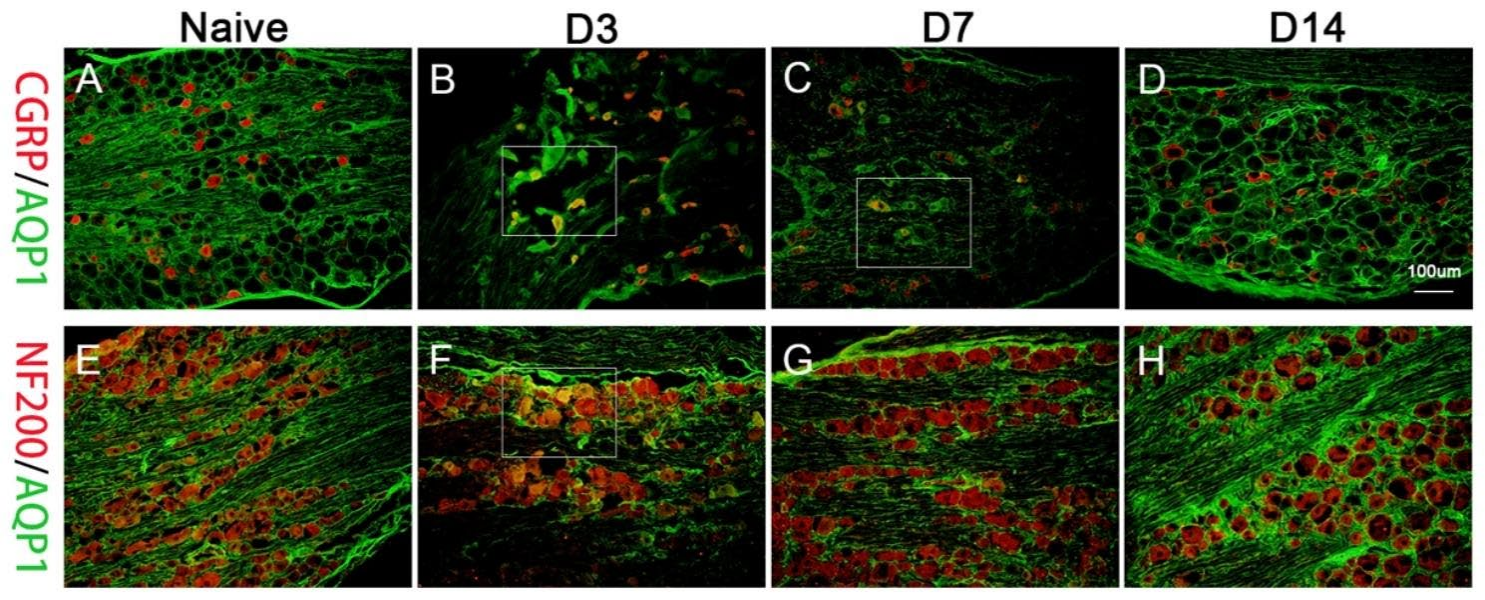
Localization of AQP1 in subneuronal cells in DRG post-SMIR. Immunofluorescence staining showed the localization changes of AQP1 in neurons before and after surgery. **(A)** Naive group, AQP1 and peptide neuronal marker CGRP had no obvious co-labeling in Naive group. **(B)** Co-labeling of AQP1 and peptide neuronal marker CGRP increased 3d after surgery. **(C)** At 7 days after surgery, there was a small amount of co-labeling between AQP1 and the peptide neuronal marker CGRP. **(D)**There was no obvious co-labeling between AQP1 and peptide neuronal marker CGRP at 14 days after surgery. **(E-H)** AQP1 and large neuron marker NF200 (co-labeling by immunization) were shown before surgery and at 3, 7, and 14 days after surgery, respectively: **(F)** AQP1 and large neuron marker NF200 were significantly co-labeling at 3 days after surgery; **(E, G, H)**: no obvious co-standard

### The effects of HIF-1α inhibitors on SMIR-induced endothelial permeability, NF200, AQP1 and pain sensitivity

On the third day post-SMIR operation, HIF-1α inhibitor (2ME2, 15 mg/kg) or equal volume DMSO was injected intraperitoneally. It was found that: Compared with the DMSO group; (A) Evans blue exudation was significantly decreased at 60 min and at 120 min after injection (*P* < 0.05). (B) PWMT was significantly increased (*P* < 0.01 or *P* < 0.001) at 30 min, 60 and 120 min after injection in SMIR group. (C) HIF-1α levels were significantly decreased after injection (*P* < 0.01). (D) AQP1 levels were significantly increased at 60 and 120 min after injection (*P* < 0.01) and (E) NF200 levels was significantly decreased at 120 min after injection (*P* < 0.01). These findings demonstrated that SMIR up-regulated HIF-1α levels and inhibited AQP1 expression, induced high permeability and axonal plasticity of BNB, significantly increased pain sensitivity.

**Fig. 5:**
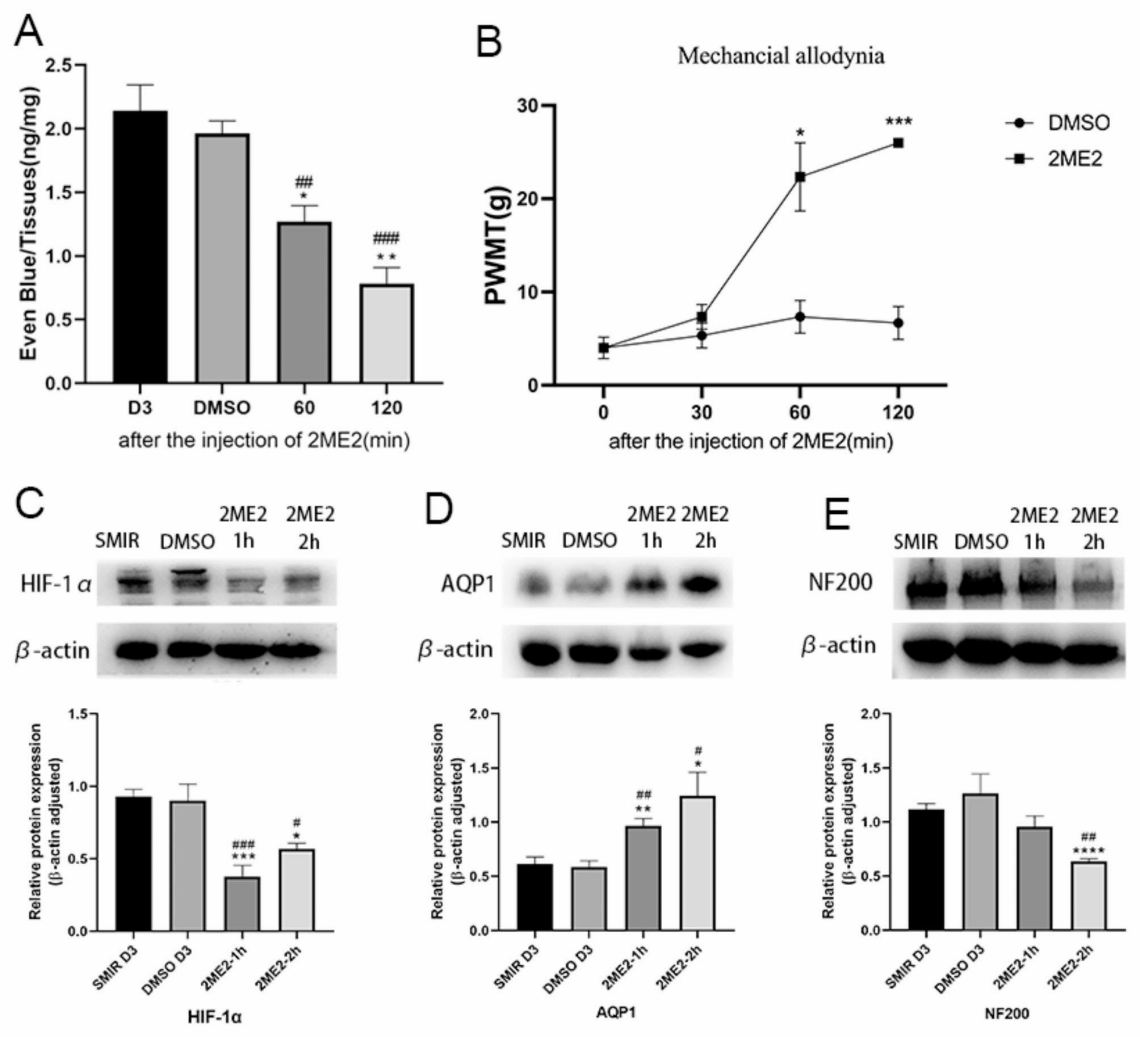
The effects of HIF-1α inhibitors on SMIR-induced endothelial permeability, NF200, AQP1 and pain sensitivity. SMIR induced high permeability and axonal plasticity of BNB through HIF-1α/AQP1, and significantly increased pain sensitivity. **(A)** Evans blue exudation was significantly decreased at 60 min and at 120 min after injection (*P < 0.05, **P < 0.01, vs. the 3rd day post-surgery group. ^##^*P* < 0.01, ^###^*P* < 0.001 vs. SMIR + DMSO group; n = 5). **(B)** PWMT was significantly increased at 60 min (*P < 0.05 vs. SMIR + DMSO group) and 120 min (***P < 0.001 vs. SMIR + DMSO group) after injection in SMIR group; n = 5. **(C)** HIF-1α levels were significantly decreased after injection (*P < 0.05, ***P < 0.001 vs. the 3rd day post-surgery group. ^#^*P* < 0.05, ^###^*P* < 0.001 vs. SMIR + DMSO group; n = 5). **(D)** AQP1 levels were significantly increased at 60 and 120 min after injection (*P < 0.05, **P < 0.01 vs. the 3rd day post-surgery group. ^#^*P* < 0.05, ^##^*P* < 0.01 vs. SMIR + DMSO group; n = 5). (E) NF200 was significantly decreased at 120 min after injection (****P < 0.0001 vs. the 3rd day post-surgery group. ^##^*P* < 0.01 vs. SMIR + DMSO group; n = 5)

## Discussion

Endoneurial microvessels control the bidirectional flow of solutes, water, nutrients, and white blood cells between blood and endoneurial Spaces, and maintain neural homeostasis. Deregulation of neural homeostasis is an early pathological event in various traumatic, immune or peripheral neuropathies [19]. Macrophages and other resident cells play an important role in inducing BNB dysfunction under pathological conditions [20]. Specific stressors induce impaired BNB function and nerve degeneration and degeneration, and endothelial cell dysfunction around peripheral nerve substructures is the initial link [21]. In this study, the BNB function test in DRG was performed using Evans blue (Fig. 2A). Results showed that extravasation was significantly increased on the 3rd and 7th days post-surgery, and the extravasation was more significant on the 3rd day post-surgery than on the 7th day post-surgery. After the 7th day, there was no further increase, but the pain sensitivity continued to increase (Fig. 1). These results suggest that SMIR triggers the release of inflammatory mediators in injured tissues and increases their recruitment through circulation and/or DRG, leading to BNB dysfunction and increased permeability. Dysregulation of neural homeostasis may be an early pathological event in the development of CPSP. This is consistent with the early pathological events of neuropathic pain reported in the literature: “Disruption of BNB leads to early sensitization of peripheral nerves and development of neuropathic pain [22]; There was a significant increase in endothelial permeability in neuron-rich areas on day 7 after nerve compression and no further increase was observed on day 7, but there was an increase in neural sensitivity [23]. “

Eutrophic inflammatory microenvironment in nerve space increases nociceptor excitability [24], BNB dysfunction leads to peripheral blood supply impairment, induces immune cell infiltration, promotes neuroinflammatory cascade and other secondary injuries [25, 26]. Different diseases cause damage to BNB, and BNB dysfunction worsens its pathophysiology [21]. These results suggest that the early stage of BNB injury is characterized by decreased effective circulating blood volume and increased tension in nerve space, leading to terminal blood supply disturbance. With the recovery of BNB function, secondary injuries of ischemia, hypoxia and ischemia reperfusion in DRG induce the reactivation of nociceptors and promote the transformation of CPSP.

AQP1 is an aquaporin widely expressed in vascular endothelium of various tissues [27], and it is a unique membrane protein of human endothelial cells [28]. AQP1 is expressed in the capillary endothelium of normal tissues and is a good marker of microvessels [29]. In this experiment (Fig. 2B), AQP1 expression decreased significantly on the 3rd and 7th day post-surgery, and decreased significantly on the 3rd day post-surgery compared with the 7th day post-surgery, contrary to the trend of significant increase of BNB permeability, indicating that SMIR-induced inhibition of AQP1 expression through water transport dysfunction may play an important role in endothelial cell and microvascular structure and dysfunction in postoperative DRG, and may be an early target for regulating BNB function.

AQP1 has been reported to be regulated by oxygen levels and downregulation of AQP1 expression was found to induce endothelial barrier hyperpermeability [30]. Decreased levels of IL-4 and IL-5 can induce the high expression of AQP1 [31, 32]. Acute hypernatremia and hyponatremia promoted the low and high expression of AQP-1, respectively [33, 34]. The results suggest that the expression pattern of AQP1 post-surgery may be related to the early dysfunction of BNB function post-surgery.

AQP1 is present in myelinated and unmyelinated Schwann cells of the sciatic nerve and plays an important role in myelin homeostasis and pain perception [35]. AQP1 is expressed on the cell body and axon plasma membrane of DRG neurons, promotes axonal growth and neuronal regeneration through water transport, regulates some types of pain perception, and is a new target for pain management [36, 37]. Microvascular barrier dysfunction impairs axonal function and intraneural edema through peripheral nerve water influx and increased hydrostatic pressure [38]. AQP1 was co-localized with neuronal marker NeuN and peptide neuronal marker CGRP on the 3rd and 7th day post-operation (Figs. 3 and 4), respectively. The density of AQP1 on the 3rd day was higher than that on the 7th day post-operation, and it was co-localized with axonal marker NF200 on the 3rd day post-operation, indicating that AQP1 was widely expressed in the neuronal body and its subcellular and it is a cellular molecule for regulating early pain perception after SMIR. Water transport dysfunction result in axonal dysfunction and nerve edema causing early disorder of postoperative nerve homeostasis. AQP1 may play an important role in the early sensitization of CPSP through the pleiotropic effect of biological activities. The peripheral inflammatory pain model upregulated HIF-1α levels [39]. Increased HIF-α stability promotes neuroinflammation [40] and microvascular hyperpermeability [41, 42], as well as improves the survival and regeneration of DRG neurons through hypoxia preconditioning protection [43]. In this study, HIF-1α was significantly increased on day 3 post-SMIR (Fig. 2C), indicating that inflammation and hypoxia microenvironment induced increased stability of HIF-α in the early stage after SMIR, which mediated the protection of cells in the DRG against hypoxia preconditioning. The expression pattern of HIF-1α may be influence early dysfunction of BNB. The AQP1 phenotype is influenced by HIF-1α levels [44]. HIF-1α regulates the expression of AQP1 induced by hypoxia in Schwann cells, which is an important mechanism of peripheral nerve edema [45]. AQP1 deficiency leads to increased stability of HIF-1α in endothelial cells [46]. The positive and negative regulation mechanism between HIF-1α and AQP1 contributes to the formation of myocardial microvascular endothelial permeability [47]. Early use of 2-methoxy estradiol (2ME2) to inhibit HIF-1α expression through AQP1 pathway, dosedependent reversal of BNB permeability after injury, showing neuroprotective effect [48]. In this experiment, 2ME2 was used 3 days post-surgery to reverse the significant decrease of AQP1 and the significant increase of barrier permeability, NF200, and hyperalgesia induced by SMIR (Fig. 5), resulting in therapeutic effects, indicating that the development of CPSP depends on the multicellular function of the neurovascular unit in the early stage post-SMIR by upregulating the level of HIF-1α and inhibiting AQP1 expression.

## Conclusion

Early BNB dysfunction mediated HIF-1α/AQP1 signal transduction activated by SMIR may be an important mechanism for promoting acute postoperative painful transformation of CPSP. Preadaptive protection of endothelial cells around nerve substructure may be an important countermeasure to inhibit CPSP transformation.

### Electronic supplementary material

Below is the link to the electronic supplementary material.

Supplementary Material 1

## Data Availability

All data generated or analyzed during this study are included in this published article.
