# Supplementary figures and images for: Blood-nerve barrier enhances chronic postsurgical pain via the HIF-1α/ aquaporin-1 signaling axis
Source: BMC Anesthesiol. 2023 Nov 21;23:381. doi: 10.1186/s12871-023-02306-7 (PMC10662690; doi:10.1186/s12871-023-02306-7)

2B-1

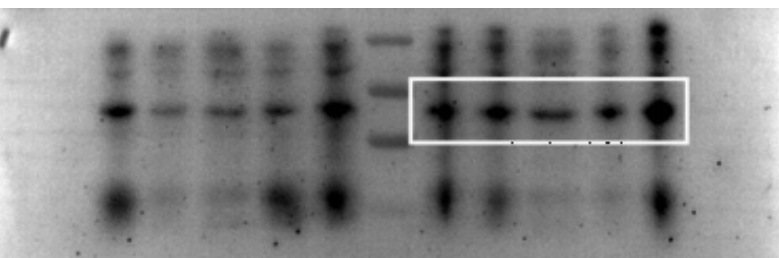

2C-1

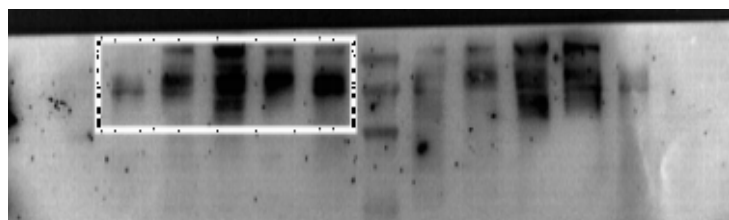

2B-2

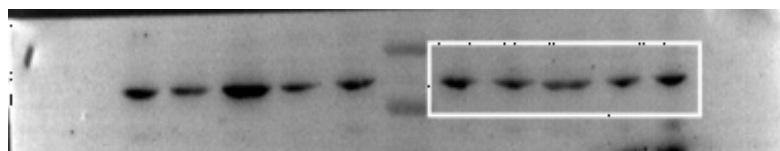

2C-2

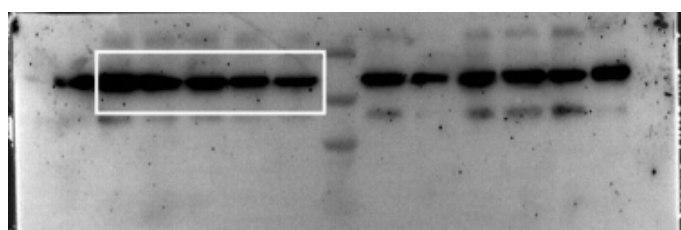

5C-1

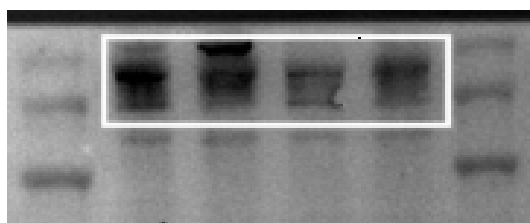

5D-1

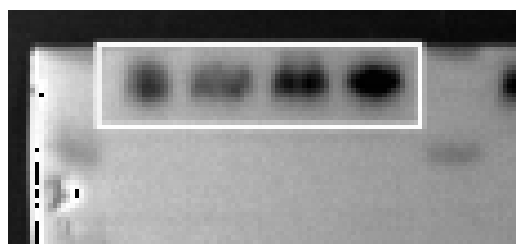

5C-2

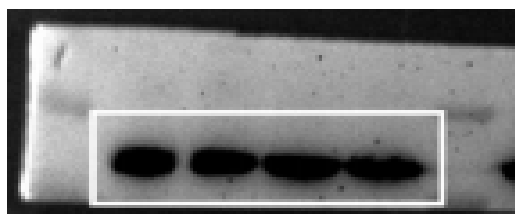

5D-2

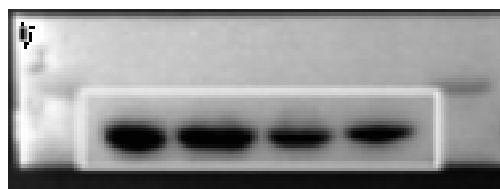

5E-1

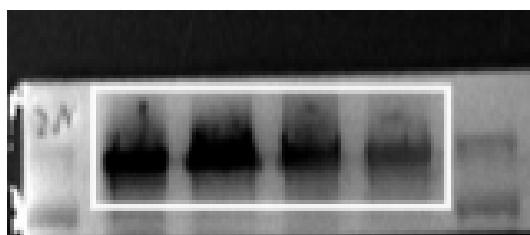

5E-2

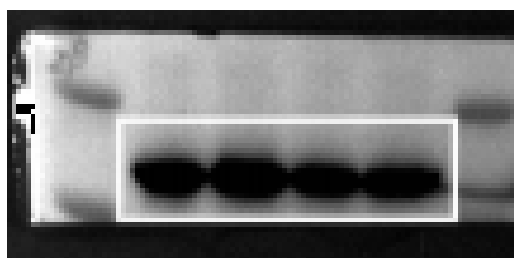

Supplement: Supplementary file 1 — Supplementary Material 1 [file 12871_2023_2306_MOESM1_ESM.pdf]
